# Expertise in evidence-based medicine: a tale of three models

**DOI:** 10.1186/s13010-018-0055-2

**Published:** 2018-02-02

**Authors:** Sarah Wieten

**Affiliations:** Philosophy Department, Indiana University of Pennsylvania, Humanities and Social Sciences Building, 505, 981 Grant Street, Indiana, PA 15705 USA

**Keywords:** Evidence-based medicine, Expertise, Evidence, Amalgamation

## Abstract

**Background:**

Expertise has been a contentious concept in Evidence-Based Medicine (EBM). Especially in the early days of the movement, expertise was taken to be exactly what EBM was rebelling against—the authoritarian pronouncements about “best” interventions dutifully learned in medical schools, sometimes with dire consequences. Since then, some proponents of EBM have tried various ways of reincorporating the idea of expertise into EBM, with mixed results. However, questions remain. Is expertise evidence? If not, what is it good for, if anything?

**Methods:**

In this article, I describe and analyze the three historical models of expertise integration in EBM and discuss the difficulties in putting each into practice. I also examine accounts of expertise from disciplines outside of medicine, including philosophy, sociology, psychology, and science and technology studies to see if these accounts can strengthen and clarify what EBM has to say about expertise.

**Results:**

Of the accounts of expertise discussed here, the Collins and Evans account can do most to clarify the concept of expertise in EBM.

**Conclusions:**

With some additional clarification from EBM proper, theoretical resources from other disciplines might augment the current EBM account of expertise.

## Background

Expertise has been a contentious concept in Evidence-Based Medicine (EBM). Especially in the early days of the movement, expertise was taken to be exactly what EBM was rebelling against—the authoritarian pronouncements about “best” interventions dutifully learned in medical schools, sometimes with dire consequences. Since then, some proponents of EBM have tried various ways of reincorporating the idea of expertise into EBM, with mixed results. However, questions remain. Is expertise evidence? If not, what is it good for, if anything?

In this work, I mean by expertise knowledge gained by subjects in the course of clinical interactions, in contrast with knowledge gained from sources such as journal articles reporting on the findings of RCTs, meta-analyses and systematic reviews or explicit medical education. As such, references to ‘experience,’ ‘expert opinion’, ‘clinical skills,’ and ‘judgment’ are all relevant to the discussion of expertise, though they emphasize different aspects of this method of knowing.

There have historically been three main models of expertise in EBM. Differences between these models, uncertainty about the difference between expertise as evidence and expertise as a force for amalgamating evidence, and a lack of consensus over whether the older models should be abandoned and replaced by the new, or used in concert, mean that the EBM conception of expertise remains impoverished. There is, however, a wealth of theoretical resources available to enrich EBM’s account of expertise. These theoretical resources come from many disciplines including philosophy, sociology, artificial intelligence, computer science, psychology and science and technology studies. Currently, these resources cannot be deployed because of the conflicting opinions within EBM about the role of expertise.

In this article, I describe the three historical models of expertise integration in EBM and discuss the difficulties in putting them into practice. I explain how all three accounts leave something to be desired, and lastly suggest that, with some additional clarification from EBM proper, theoretical resources from other disciplines might augment the current EBM account of expertise. I conclude the paper by recommending one of these resources, the Collins and Evans account of expertise with roots in Science and Technology Studies, as the most useful for augmenting the current EBM conception of expertise.

Of course, all three are just models—there is reason to believe that none of this is precisely what happens in EBM with regard to expertise in practice. Still, to understand a movement, it is important to consider not just what the movement does, but what it takes itself to be attempting to accomplish. A principle of charity compels us to look at EBM’s theoretical accounts of expertise, even if these accounts are imperfectly implemented.

## Main text

The first model for expertise in EBM was articulated in EBM’s 1992 “debut” article, “Evidence-Based Medicine: A New Approach to Teaching the Practice of Medicine. [1]” While proponents of EBM often point to historical precursors, this article began the modern movement. In this article, the Evidence Based Working group wrote, “Evidence-based medicine *de-emphasizes intuition, unsystematic clinical experience*, and pathophysiologic rationale as sufficient grounds for clinical decision making and stresses the examination of evidence from clinical research [1].^1^” Of these three targets for de-emphasis, two, intuition and unsystematic clinical experience, might be seen as related to expertise. The third, pathophysiologic rationale, involves using evidence about mechanisms or causation in order to select interventions for use.

From this article arose the first model of expertise in EBM.^2^ This pyramid shaped model is a familiar symbol of EBM although other non-pyramid shaped hierarchies have since been developed within the movement. The pyramid is divided horizontally into a series of layers. The labeling of the layers varies from depiction to depiction, but the methods of creating evidence at the top of the pyramid are considered to be more valuable and trustworthy than those at the bottom. The portion at the very top of the pyramid is usually taken to represent randomized controlled trials (RCTs), the method of choice for evidence production in EBM, or in later versions, various amalgamations or overviews of RCTs, such as meta-analyses or systematic reviews. Below these are other kinds of studies which lack blinding or randomization, often called observational studies. Below these, at the very bottom of the pyramid, in the lowest position, is “background information” or “expert opinion” or “clinical expertise. [2]”.

**Fig. 1:**
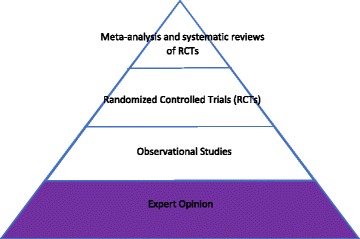
Is based on written information in EBMWG, 1992

There are several interesting things about this model. For all the talk in the debut article about de-emphasizing concepts related to expertise, expertise does show up within the pyramid as a kind of evidence, albeit one with very low standing. In this way, the pyramid model keeps expertise internal to evidence. In addition, the pyramid shape itself can tell us something. This pyramid operates very differently from the other famous pyramid model—the food pyramid. In the food pyramid, the smaller triangle at the top of the pyramid is the substance to be avoided—that is, this area is the sugar and salt area and public health officials are warning us that we *ought not* to consume too much from this group. In contrast, in the EBM pyramid, the top portion is composed of RCTS and amalgamations of RCTs, which are of “highest quality” according to EBM; that is, it is not telling us not to do too many RCTs, but rather that they, despite being of the “highest quality,” make up the minority of studies done.^3^ Understanding the pyramid structure also tells us that according to Fig. 1, expertise is the most common type of evidence, even if it is not of the highest quality according to EBM.

The GRADE model, though not pyramid shaped, is an update of this first kind of model, so I include it as a type of Fig. 1. GRADE, Grading of Recommendations Assessment, Development and Evaluation, is a system for ranking evidence created by a group at Oxford in 2000. While GRADE is similar to the traditional pyramid in certain ways, such as placing RCTs and the various ways of amalgamating them at the top of the ranking and expertise at the bottom, it does add one additional component. In GRADE, there is an additional vector along which studies are measured—the quality with which they are carried out. This additional vector of quality allows for a more complete assessment of the balance of benefits and harms suggested by studies of an intervention. So, while RCTs and amalgamations of RCTs are given a high original score and observational studies a low one on the basis of methodology, GRADE leaves open the possibility that a very well carried out observational trial might end with a higher score than an RCT or amalgamation of RCTs of very low quality [3].

The creators of the GRADE system had this to say about expertise: “Systems that classify “expert opinion” as a category of evidence also create confusion. Judgment is necessary for interpretation of all evidence, whether that evidence is high or low quality. Expert reports of their clinical experience should be explicitly labelled as very low-quality evidence, along with case reports and other uncontrolled clinical observations [3]”. This description illuminates what will be a continual problem for expertise in EBM—is expertise a kind of evidence, or something external to evidence? For GRADE, expertise is allowed some kind of evidence-external role: the role of judgement, whatever that might be. However, GRADE keeps with the pyramid tradition, also counting expertise as a kind of low quality evidence. GRADE retains most of the hierarchy in place in the pyramid style models but adds considerations of quality to the equation and makes a distinction about roles for expertise *as* evidence and *as external to* evidence, making it a slightly modified version of Fig. 1.

**Fig. 2:**
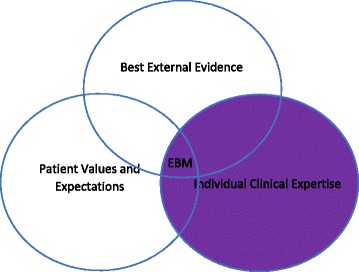
Is based on written information in Sackett et al. [2]

A second model comes out of the clarificatory work of Sackett et al., in their 1996 work, “EvidenceBased Medicine; what it is and what it isn’t [4].”^3^ They sought to clarify, among other things, the role of clinical expertise in EBM, given that there had been some clinician push back to the hardline stance taken in the debut article in 1992 [5–9]. They write, “By individual clinical expertise, we mean the proficiency and judgment that individual clinicians acquire through clinical experience and clinical practice. Increased expertise is reflected in many ways, but especially in more effective and efficient diagnosis and in the more thoughtful identification and compassionate use of individual patients’ predicaments, rights, and preferences in making clinical decisions about their care [4]”. This new definition suggests that the new model will be interested, not just in the evaluation of evidence, but also in the application of evidence and other activities. This definition of expertise couches it almost entirely in terms of roles that are external to evidence. Expertise on this account is about efficient reasoning skills for diagnosis, and proper application of evidence to individual patients’ needs and values. This is in line with Fig. 2 of expertise that was developed from this paper and others written by Sackett around this time [10]. Figure 2 features a Venn-diagram structure with three overlapping circles of influence: evidence, clinical expertise, and patient values and preferences. The intersection of all three is labeled “EBM.”

This new model suggests a few things about the re-defined role for expertise in EBM. First, in this model, expertise is a separate category from evidence, since each has its own separately labeled circle of influence. This is in contrast to Fig. 1, in which expertise was taken to be a kind of evidence, if a low-quality one. In addition, it resolves the confusion created in the GRADE version of Fig. 1, in which it expertise was sometimes considered internal to evidence and sometimes was considered to be external to evidence. This model also brings in an additional consideration, patient values and preferences, as its own circle of influence. Given that the definition of expertise suggests that an important part of expertise is responding to patient values and preferences, it is interesting that these are still depicted as discrete components. Perhaps surprisingly, both the description of this model and the way it has been pictorialized suggest that equal consideration is to be given to all three components—earlier accounts might have lead us to expect an emphasis on the evidence component. In addition, the examples of expertise used in this definition suggest what some of the concrete extraevidentiary roles for expertise in EBM might be—diagnostic reasoning and recognition and the application of population based evidence to particular patients.

**Fig. 3:**
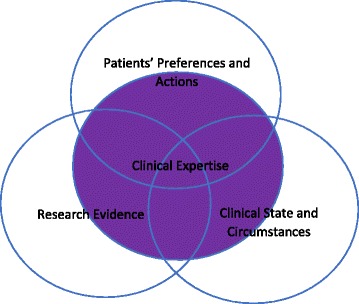
is based on written information in Haynes 2002. Imagines very similar to these appear in Howick 2011

The third and most recent model of the role of expertise in EBM comes from the 2002 Haynes.

et al. article, “Clinical expertise in the era of evidence-based medicine and patient choice [11]”.^4^ This model retains the circles of influence format of Fig. 2 but modifies the content of the circles of influence and the relationship of expertise to these circles of influence. In Fig. 3, the three circular components are “research evidence,” “patients’ preferences and actions,” and “clinical state and circumstances.” I will not say much about “research evidence” and “patients’ preferences and actions” as they seem to largely line up with those components in Fig. 2.

The “clinical state and circumstances” are explained by Haynes et al. by writing,

Patients’ clinical state, the clinical setting, and the clinical circumstances they find themselves in when they seek medical attention are key, and often dominant, factors in clinical decisions. For example, a patient with an undiagnosed symptom cannot be readily moved from a diagnostic decision to a therapeutic decision. Furthermore, people who find themselves in remote areas when beset by crushing retrosternal chest pain may have to settle for aspirin, whereas those living close to a tertiary care medical centre will probably have many more options — if they recognise the symptoms and act promptly! Similarly, a patient with atrial fibrillation and a high bleeding risk, as with the patient described at the beginning of this editorial, may experience more harm than good from anticoagulation treatment, whereas a patient with a high risk for stroke and a low risk for bleeding may have a substantial net benefit from such treatment [11].

This new third component includes additional considerations of the clinical situation that may prevent the direct application of evidence to particular patients. Encircling portions of all three of these circular components is a fourth oval component labeled “clinical expertise.”

Haynes and co-authors write of this expanded role of expertise in EBM saying,

Clinical expertise includes the general basic skills of clinical practice as well as the experience of the individual practitioner. Clinical expertise must encompass and balance the patient’s clinical state and circumstances, relevant research evidence, and the patient’s preferences and actions if a successful and satisfying result is to occur. Accomplishing this goal often involves sorting through tradeoffs. Clinicians must be atop not only the research evidence, but they must also acquire and hone the skills needed to both interpret the evidence and apply it appropriately to the circumstances — doing the right things. Finally, although communication with patients has always been important, determining the role in decision making that patient’s desire, ascertaining their preferences, and providing patients with the information they need to make an informed choice has never been more challenging [11].

This passage rehearses some of the same roles for expertise that we heard in the GRADE version of Fig. 1. Clinical expertise is internal to evidence, in that expertise in some way constitutes a kind of evidence, but mostly expertise is external to evidence. These external activities include diagnosis, prognosis, effective patient communication, the correct performance of a treatment or test, and the application of population-based evidence to particular individual patients. What differs is how this expertise is structured. Instead of expertise being its own domain, its own subject which needs to be properly combined with research evidence and patient preferences to get EBM as in Fig. 2, expertise is the force which amalgamates a different set of components (research evidence, patient preferences and values and the clinical state and circumstances) together. Rather than a component itself, it is the force that adjudicates between the other components, weighing and balancing the requirements imposed by each component.

While the changes in models might indicate that EBM is more open to including a role for expertise in their movement, this sequence of models leaves us with many questions. Which model should we use? Should we assume that each model was meant to replace the one that came before, meaning we should at this point only be concerned with Fig. 3? Or should we instead attempt to take all three into consideration? It is still unclear if expertise in EBM is to be taken as internal or external to evidence. In Fig. 1 expertise is considered to be internal to evidence, though it is not ranked very highly as evidence. In the later GRADE version of Fig. 1, expertise is seen as both internal and external to evidence. In Figs. 2 and 3 expertise is largely considered to be external to evidence. Then there is the issue of conflict between components—when different sorts of evidence suggest different courses of action or patient preferences are not in line with the research evidence, what should we do? In Fig. 1, the hierarchy implicit in the model tells us which evidence is to be preferred in case of a conflict. In Fig. 3, expertise seems to be the force that is supposed to make these kinds of judgements, but it is not clear how. In Fig. 2 there is no kind of guidance at all about how these kinds of conflicts are to be worked out. A final concern presents itself when we consider medical education. On all accounts, expertise is to have an important role in EBM. However, authoritarian experience as taught without warrant and with poor outcomes for patients in medical schools was, in the accounts of many of the founders of EBM, their main motivation for the movement.^5^ Given this desire to both integrate expertise and replace it in medical education, it becomes important to have an account of well-done expertise. What does this “good expertise” look like? How can we differentiate it from the kind of excesses that first inspired evidence-based medicine? And once we know this, can we teach it to medical students? How?

Luckily, EBM is not the only movement and medicine not the only discipline where issues about expertise have come to the forefront. Fields like artificial intelligence, philosophy, cognitive science, epistemology, science and technology studies, sociology, psychology and biostatistics all have developed literatures in the study of expertise. In the reminder of this paper I will consider what a selection of these literatures have to offer to the EBM problems with expertise, focusing on the work of Turner, Collins and Evans, Dreyfus, and Bishop and Trout.^6^

Of course, most of this research cannot be utilized without some input from EBM itself. For example, some of this literature is incompatible with an entirely external to evidence account of expertise. In order to know if the resources of an account might be useful to EBM, we need to know where EBM stands on a number of questions. Perhaps in time, manuals for how to practice EBM will themselves include more developed accounts of EBM which answer some of these questions. This will make clear which of these literatures could be meaningfully mobilized for use in EBM.

A first possibility for an account of expertise that might be useful to EBM is provided by the philosopher and artificial intelligence critic Hubert Dreyfus in works like “Mind Over Machine” and “What Computers Still Can’t Do [15, 16]”. It has been suggested that his account of expertise might be useful for EBM because of its uptake in the field of nursing, especially in the work of Patricia Benner [17]. In this account, expertise is a “flow of skilled coping,” an unconscious state of movement from one competent activity to the next. Favored examples are driving a car, playing basketball or chess, and performing nursing duties. In the Dreyfus account, expertise plays roles that are both internal and external to evidence. This account strongly resists attempts to formalize or rationalize expertise, but does suggest what to look for in a good expert: un-reflective certainty about what needs doing next and a lack of dependence on explicit rules for functioning. While the Dreyfus account remains somewhat antithetical to components of EBM [18], if adopted it would provide an answer to the question of expertise as internal/external to evidence, and provide very clear pedagogical resources for developing new experts.

A second possibility is the work of philosopher and sociologist Stephan Turner. In Turner’s account of expertise, we should listen to experts because of the political power granted to them by society, not necessarily because of any particular rare epistemic status they may hold [19, 20]. The reason a layperson should defer to the knowledge of the expert is because laypeople in general have conferred power on experts whom they trust, although this trust is not equally placed in all experts. This account is very much in line with the kind of authoritative expertise that EBM originally took as its target; the goal of EBM was to put true knowledge in the place of unchallenged received wisdom based on power. Indeed, when asked for their motivation for creating EBM, many of the movement’s early proponents pointed to incidents in their medical education in which they felt that their teachers were exercising expertise in this fashion (i.e. as an arbitrary abuse of power, rather than an expression of skilled familiarity or a privileged epistemic perspective). If EBM were to adopt Turner’s power based account of expertise, this would be in line with Fig. 1, in which expertise, while internal to evidence, is its lowest expression. While this is the oldest model, it is evident that this is still how some EBM practitioners conceive of expertise, based on expressions such as the perennial popularity of the GOBSAT [Good Old Boys Sit Around A Table] joke [21]. Pedagogy based on Turner’s account might (cynically) include advice on how best to inspire trust in the public such that the public will confer power on you, or perhaps (less cynically) how to wield such power as benevolently as possible. However, adopting the Turner account seems to entail ignoring Figs. 2 and 3, which take a more reconciliatory approach to expertise at least in roles that are external to evidence. While it is possible that these two newer models are in error, it would require a strong explanation for backing away from this reconciliatory view on the part of EBM proponents to adopt the Turner account.

An additional option to consider is the account of expertise in Michael Bishop and J. D. Trout’s 2004 book, “Epistemology and the Psychology of Human Judgement [22]”. For Bishop and Trout, human expertise or intuition is just the unaided gut feelings of experts, the baseline against which their preferred set of decision aids, Statistical Prediction Rules (SPRs), need to perform in order to be considered effective. The book illustrates the myriad ways in which this intuition fails to get the “right” answer in many situations, although it is not always clear how the “right” answers that these two methods are being measured against were themselves obtained. While it is not terribly clear on this account if they consider expertise to be internal or external to evidence, since they do not discuss many of the traditionally cited external roles for evidence and seem to think that in cases where intuition is used, it is the only cause of particular decisions being made, that their outlook is closer to an internal to evidence conception of expertise. In so far as they are unimpressed with the quality of expertise as a reasoning strategy, their account is compatible with Fig. 1, in which expertise is internal to evidence, but is of very low quality. However, adopting the sort of account of expertise that Bishop and Trout suggest would make a mystery of more recent attempts by EBM to integrate expertise and investigate the external to evidence roles of expertise. Just like the Turner model, accepting the Bishop and Trout model of expertise would simplify the discussion about expertise in EBM (and their interest in algorithmic decision-making tools might be in line with other components of EBM), but would require the rejection of this more recent and reconciliatory work on expertise in EBM.

A final option to consider here (although there are others worth addressing) is the work of Harry Collins and Robert Evans in their book “Rethinking Expertise” in Science and Technology Studies [23]. Although the Collins and Evans account comes from a sociologically influenced discipline, they are quick to distinguish themselves from the kind of account given by Turner, which they call “relational.” Instead, they begin with realism about expertise. Collins and Evans think there are at least two important kinds of expertise: interactional and contributory expertise, although they supply a more detailed “periodic table” of expertise as well. Contributory expertise is held by those who know enough about a domain to make an original contribution to that domain. Interactional expertise is “the mastery of the language of a domain, and mastery of any language, naturally occurring or specialist, requires enculturation within a linguistic community [23]”. Interactional expertise requires that a would-be expert is able to discuss the details of a particular domain so well that they are not conversationally separable from a contributory expert, though they do not in fact know enough to make an original contribution to the domain in question. Indeed, Collins and Evans suggest the use of Turing-style tests in conversation with contributory experts to determine who is an interactional expert.

## Conclusions

The realism about expertise that underpins the Collin and Evans account fits nicely with the reconciliation with expertise in EBM in Figs. 2 and 3, but would be an odd fit with Fig. 1. Additionally, the interactional/contributory distinction might be helpful in EBM as a way of disentangling the kind of expertise that is held by clinicians hoping to use EBM principles, from researchers who are hoping to develop or refine those principles. This distinction could also assist in making pedagogical goals for each group clearer. But while at first it might seem that the interactional/contributory distinction maps onto and provides clarity to the issue of whether expertise is internal/external to evidence, on closer look these remain separate issues. The Collins and Evans account of expertise could add a great deal to the discussion of expertise in EBM, but not without additional clarifications from within EBM.

The previous section has provided some suggestion of the resources for fleshing out and improving the currently confused EBM account of expertise. While there are many solutions available, I have shown that these solutions cannot be marshaled by EBM without some decision making from EBM, about considering expertise as internal or external to evidence among other things. That being said, I recommend the Collins and Evens concept of expertise as the most useful augmentation of expertise in EBM because of its “realist” stance on expertise, the usefulness of its interactional/contributory distinction, and its pedagogical bent.

## Abbreviation

EBM: Evidence-Based Medicine

## Footnotes

1 Emphasis mine.

2 See the Appendix for a diagram of this model

3 See the Appendix for a diagram of this model

4 See the Appendix for a diagram of this model

5 [After my “conversion” to EBM] “I became a ‘trouble-maker,’ constantly questioning conventional therapeutic wisdom, and offending especially the sub-specialists when they pontificated (I thought) about how I ought to be treating my patients. I had a stormy time in obstetrics, where I questioned why patients with severe pre-eclampsia received intravenous morphine until their respirations fell below 12 per minute. I gained unfavorable notoriety on the medical ward, where I challenged a consultant’s recommendation that I should ignore my patient’s diastolic blood pressure of 125 mmHg ‘because it was essential for his brain perfusion. And I deeply offended a professor of pediatrics by publicly correcting him on the number of human chromosomes (they had fallen from 48 to 46 the previous month!) [12].” “It made me wonder whether what I had been taught at medical school might have been lethally wrong, at least in the circumstances in which I was working, and precipitated a now incurable “septicemia” about authoritarian therapeutic prescriptions and prescriptions unsupported by trustworthy empirical evidence [13]”. “For R.B.H. [Haynes] the need for an additional basic science for clinical medicine entered his consciousness during a pre-clerkship lecture on Freudian concepts of psychiatric illness. When he meekly requested the evidence for one of these concepts, the speaker expostulated that the purpose of the lecture was to transmit content, not defend it (and he admitted that he didn’t believe it himself). This need for a more systematic approach to gathering and interpreting clinical evidence was reinforced repeatedly during R.B.H.’s early postgraduate training in eastern Canada, most noisily in a running battle between two of his senior attending as to how he was to measure blood pressures of their patients [14].”

6 Some proponents of EBM, especially those for whom the point of the movement was to champion RCT methodology, may be surprised at my suggestion that these theoretical resources might help EBM. Given that the problem seems to be a conceptual/theoretical tangle with practical implications, I assume it is possible that theory will be needed to solve the problem and mitigate the implication.
